# Preparation of bioactive peptides with antidiabetic, antihypertensive, and antioxidant activities and identification of α‐glucosidase inhibitory peptides from soy protein

**DOI:** 10.1002/fsn3.1038

**Published:** 2019-04-15

**Authors:** Rongchun Wang, Hongxing Zhao, Xiaoxi Pan, Caroline Orfila, Weihong Lu, Ying Ma

**Affiliations:** ^1^ Department of Food Science and Engineering, School of Chemical Engineering and Chemistry Harbin Institute of Technology Harbin China; ^2^ School of Food Science and Nutrition University of Leeds Leeds UK

**Keywords:** ACE inhibitory activity, antidiabetic activity, antioxidant activity, peptide, soy protein, α‐glucosidase inhibitory activity

## Abstract

In this study, the peptides of soy protein obtained by enzymatic digestion with proteases were analyzed for their antidiabetic, antihypertensive, and antioxidant activities. Peptides prepared with alkaline proteinase (AP) exhibited the highest α‐glucosidase inhibitory activity compared with those from papain and trypsin digestion. AP hydrolysates also exhibited dipeptidyl peptidase IV (DPP‐IV) inhibitory, angiotensin‐converting enzyme (ACE) inhibitory, and antioxidant activities. Gastrointestinal digestion of peptides enhanced α‐glucosidase, DPP‐IV, and ACE inhibitory activities compared with AP hydrolysates. AP peptides showing highest α‐glucosidase inhibitory activity were purified by anion‐exchange and size‐exclusion chromatography， and identified using tandem MS. We found three novel α‐glucosidase inhibitory peptides with sequences LLPLPVLK, SWLRL, and WLRL with IC_50_ of 237.43 ± 0.52, 182.05 ± 0.74, and 162.29 ± 0.74 μmol/L, respectively. Therefore, peptides hydrolyzed from soy protein are promising natural ingredients for nutraceutical applications assisting in the management of diabetes.

## 1 INTRODUCTION

Diabetes mellitus (DM) is a common chronic metabolic disorder characterized by dysregulation of blood glucose and has been considered as a major global health issue now and in the future. DM presents in two main forms: Type 1 insulin‐dependent DM (T1DM) constitute 10% of all DM cases, and type 2 non‐insulin‐dependent DM (T2DM) constitute 90% of all DM cases. It is predicted that the number of patients with diabetes will increase from 350 million today to 592 million by 2035 (Lee et al., 2016). Management of T2DM normally involves lifestyle modification and pharmacologic therapy. Several drugs, which are approved to treat hyperglycemia in T2DM, are costly and have serious side effects. For example, glucagon‐like peptide‐1 (GLP‐1) receptor agonists have been shown to increase the risk of kidney injury (Qaseem et al., 2017). The serious side effect and the toxicity associated with some therapeutic drugs indicate the demand for diet‐derived antidiabetic agents or approaches that are considered natural and safe. Enzymes associated with the regulation of postprandial hyperglycemia, such as α‐glucosidase, α‐amylase, and dipeptidyl peptidase IV (DPP‐IV), have been recognized as therapeutic target. α‐glucosidase is a membrane‐bound enzyme located in the epithelium of the small intestine, which catalyses the cleavage of glucose from disaccharides (Matsui, Yoshimoto, Osajima, Oki, & Osajima, 1996). Inhibition of this enzyme has been recognized as an effective approach for lowering serum glucose level (Johnson, Lucius, Meyer, & Gonzalez De Mejia, 2011). α‐glucosidase, which catalyses the reaction from dextrin to glucose in the small intestine, plays a key role in the digestion and absorption of carbohydrates. Thereby, α‐glucosidase inhibitors such as acarbose, voglibose, and miglitol effectively diminish postprandial hyperglycemia by impeding the digestion of carbohydrates. However, these types of inhibitors have unpleasant and potentially serious gastrointestinal side effects which limit use. Other therapeutic targets include dipeptidyl peptidase IV (DPP‐IV), which is known to cause the inactivation of incretin hormones GLP‐1 and gastric inhibitory peptide (GIP) (Drucker, 2007). By inhibiting DPP‐IV, it is possible to increase the half‐life of GLP‐1 and GIP resulting in enhanced insulin secretion (Nongonierma & FitzGerald, 2013). Furthermore, according to the National Diabetes Statistic Report of USA in 2017, about 73.6% of adults with DM have hypertension, which increases the risk of coronary heart disease (Abuissa, Jones, Marso, & O'Keefe, 2005; CDC, 2017), peripheral artery disease, stroke, and even kidney disease. There is also growing evidence indicating that the development of diabetes is related to oxidative stress (Asmat, Abad, & Ismail, 2016). Therefore, it is significant for identifying diet‐related strategies that could address these different pathologies.

Bioactive peptides have a wide range of functional properties, including antimicrobial, antihypertension, hypoglycemic activity, immunomodulation, and antioxidative functions. Due to their lower relative molecular weight, higher absorption, and bioavailability (Koopman et al., 2009), food‐derived bioactive peptides have shown potential to serve as natural alternatives or complements to synthetic drugs. Many studies have demonstrated hypoglycemic effects of bioactive peptides from hemp seed protein, goby fish, and egg‐yolk protein (Nasri et al., 2015; Ren et al., 2016; Zambrowicz et al., 2015).

The objective of this research was to prepare peptides from soy protein hydrolysates with enzymolysis and to screen bioactive peptides with high α‐glucosidase inhibitory activities. Successive separation and purification were carried out, and the α‐glucosidase inhibitory peptides were identified. We also measured the in vitro antioxidative activity and the angiotensin‐converting enzyme (ACE) inhibitory activity of the soy protein hydrolysates. The potential of soy bioactive peptides as ingredients for nutraceutical or functional food formulation is discussed.

## 2 MATERIALS AND METHODS

### 2.1 Materials

Soy protein powder was purchased from Harbin Gaoke Group Co., Ltd. Enzymes including alkaline proteinase (200 U/mg), papain (800 U/mg), trypsin from porcine pancreas (250 U/mg), pepsin from porcine gastric mucosa (500 U/mg), and pancreatin from porcine pancreas were purchased from the Summus Chemical Company; 4‐nitrophenyl‐α‐D‐glucopyranoside (PNPG), α‐glucosidase from *Saccharomyces cerevisiae*, acarbose hydrate; dipeptidyl peptidase IV (DPP‐IV) from human; gly‐pro‐*p*‐nitroanilide, hippuryl‐l‐histidyl‐l‐leucine (HHL), angiotensin I‐converting enzyme (ACE) from rabbit lung (2.0 U/mg); and hippuric acid (HA), DEAE‐52, and gel Sephadex G‐15 were obtained from Sigma‐Aldrich. All other chemicals and reagents were of analytical grade and commercially available.

### 2.2 Preparation of soy protein peptides

The protein content of the raw material was determined by using the Kjeldahl method. Preparation of the protein hydrolysates was performed in a temperature‐ and pH‐controlled 500‐mL reaction vessel equipped with a stirrer. Three grams of soy protein powder was dispersed in distilled water to obtain 2% protein slurry (w/v). Subsequently, the protein slurry was heated to 100°C for 10 min for denaturation. After cooling down, 0.5 mol/L sodium hydroxide (NaOH) and 0.5 mol/L hydrochloric acid (HCl) aqueous solutions were used to adjust the pH of the slurry to the optimum conditions recommended by the manufacturers for each protease: AP (pH 9, 50°C), papain (pH 6.5, 60°C), and trypsin (pH 7, 37°C). The protease (6,000 U/g, on the basis of the protein content of slurry) was added to initiate the protein enzymatic hydrolysis. Temperature and pH values were kept constant until the degree of hydrolysis no longer changed. Then, the hydrolysates were heated in a boiling water bath for 10 min to inactive the protease. After removal of the precipitate by centrifugation (4,436 *g*, 10 min), it was collected supernatant and stored at −20°C until use. The degrees of hydrolysis (DH) values were measured by “pH‐stat” method described by Adler‐Nissen (1986).

### 2.3 Molecular weight distribution

The molecular weight distributions of hydrolysates were evaluated by size‐exclusion HPLC (Waters) on a TSKgel 2000 SWxl column (7.8 mm × 300 mm). One hundred milligram of hydrolysate powder was dissolved in 10 ml mobile phase (acetonitrile/water/trifluoroacetic acid, 40/60/0.1), sonicated for 5 min, centrifuged at 8,000 rpm for 10 min, and then filtered through the microporous membrane. The injection quantity was 10 μl with the flow rate of 0.5 ml/min. The effluent was monitored by UV detector at 220 nm. Cytochrome C (MW: 12,384 Da), bacitracin (MW: 1,422 Da), leupeptin (MW: 427 Da), and tryptophan (MW: 204 Da) were used as molecular weight standards.

### 2.4 α‐glucosidase inhibitory activity assay

α‐glucosidase inhibitory activity assay was performed according to a method by Kim with slight modifications (Kim, Wang, & Rhee, 2004). The α‐glucosidase inhibitory rate of the hydrolysate was estimated in a 96‐well microplate using a MR‐96A microplate reader (Mindray) by monitoring the amount of 4‐nitrophenol (PNP) released from 4‐nitrophenol‐α‐D‐glucopyranoside (PNPG). A dose (50 μl) of sodium phosphate buffer (pH 6.8, 0.2 M), 50 μL of PNPG (1 mg/ml) dissolved in sodium phosphate buffer, and 50 μl of the samples dissolved in the same buffer were mixed adequately in a 96‐well microplate. Then, it was incubated for 5 min at 37°C, and the reaction started immediately followed by the addition of 10 μl of α‐glucosidase solution (0.2 U/ml of the buffer). The plate was incubated for another 30 min at 37°C, and then, the reaction was terminated by the addition of 80 μl of 1 M Na_2_CO_3_ solution. The absorbance of released product PNP was measured immediately at 405 nm. Sodium phosphate buffer and acarbose (10 mg/ml of the buffer) were used as the negative and positive control, respectively. The percentage of α‐glucosidase inhibition was calculated as follows:

(1) $$\alpha -\text{glucosidase inhibition rate}\left(\%\right)=\frac{{\text{OD}}_{\text{A}}-{\text{OD}}_{\text{S}}}{{\text{OD}}_{\text{A}}-{\text{OD}}_{\text{B}}}\times 100$$

where OD_A_ is the absorbance of the supernatant in which the sample is replaced by the same amount of buffer; OD_S_ is the absorbance of the supernatant with the tested sample; and OD_B_is the absorbance of the supernatant in which the tested sample and α‐glucosidase solution are replaced by the same amount of buffer. IC_50_ value was obtained and used to evaluate the α‐glucosidase inhibition rate of the sample, which was calculated as the concentration of the tested sample required for α‐glucosidase inhibition rate of 50%.

### 2.5 Dipeptidyl peptidase IV (DPP‐IV) inhibition assay

DPP‐IV inhibition assay was according to the method from Harnedy et al with minor modifications (Harnedy, O'Keeffe, & Fitzgerald, 2015). The assay was performed in a 96‐well microplate; 25 μl of test sample was preincubated with 25 μl of substrate gly‐pro‐*p*‐nitroanilide (12 mM) at 37°C for 10 min, after added 50 μl of DPP‐IV (0.02 U/ml of Tris‐HCl buffer, pH 8.0); the mixture was incubated at 37°C for 30 min; and then, 100 μl acetic acid–sodium acetate (1 M) was added to terminate the reaction. The DPP‐IV inhibition rate (%) was calculated as following:

(2) $$\text{DPP}-\text{IV inhibition rate}\left(\%\right)=1-\frac{{\text{OD}}_{\text{S}}-{\text{OD}}_{\text{N}}}{{\text{OD}}_{\text{A}}-{\text{OD}}_{\text{B}}}\times 100$$

where OD_A_ is the absorbance of the supernatant in which the tested sample was replaced by the same amount of buffer; OD_S_ is the absorbance of the supernatant with the tested sample; OD_B_ is the absorbance of the supernatant in which the tested sample and DPP‐IV solution are replaced by the same amount of buffer; and OD_N_ is the absorbance of the supernatant in which the DPP‐IV solution is replaced by the same amount of buffer.

### 2.6 ACE inhibition assay

The determination of ACE inhibitory activity was performed by using in vitro method described by Wu and Ding with slight modifications (Wu & Ding, 2002). This assay was based on the release of HA from HHL with ACE enzymolysis. For the assay, 200 μl ACE (0.1 U/ml 0.1 M borate buffer containing 0.3 M NaCl at pH 8.3) was added to the sample (100 μl), and the mixture was incubated at 37°C for 10 min. Then, 200 μl of HHL (5 mM in 0.1 M borate buffer containing 0.3 M NaCl at pH 8.3) was added into each well as substrate. The enzymatic reaction subsequently carried out at 37°C for 60 min. The reaction was terminated by adding 300 μl of 1 M HCl. Borate buffer was used as the control. The final HA was extracted with 1.5 ml of ethyl acetate; after 90°C of incubation, the organic layer would be dry and dissolved in 2 ml of distilled H_2_O. The amount of HA produced was determined by an HPLC system. An aliquot of 50 μl from the reaction mixture was analyzed by an HPLC system equipped with Waters C18 column (ODS, 150 × 4.6 mm, 5 μm particle size) using an elution of formic acid (0.4%): methanol (3:7 v/v) at a constant flow rate of 0.5 ml/min, and HA was detected by UV detector at 228 nm. The ACE inhibition rate (%) was calculated as follows:

(3) $$\text{ACE inhibition rate}\left(\%\right)=\frac{A-C}{A-B}\times 100$$

where *A* is the chromatography peak area of the supernatant in which the tested sample is replaced by the same amount of buffer; *C* is the chromatography peak area of the supernatant with tested sample; and *B* is the chromatography peak area of the supernatant with no tested sample and ACE solution.

### 2.7 Antioxidative activities of soy protein peptides

#### 2.7.1 Hydroxyl radical scavenging ability

The antioxidant activity of the soy protein peptides was determined by a hydroxyl radical scavenging assay involving in the Fenton reaction, according to Hanasaki (Hanasaki, Ogava, & Fukui, 1994). The samples were prepared into 0.1, 0.5, 2, 5, and 10 mg/ml. Then, 1 ml of 9 µmol/L FeSO_4_ solution and 1 ml of salicylic acid–ethanol solution (9 µmol/L，50% ethanol solution) were added respectively and mixed well, with the addition of 1 ml of 8.8 µmol/L H_2_O_2_ (0.03%) reaction initiated. The mixture was incubated in a 37℃ water bath for 30 min and then centrifuged at 2,896 *g* for 5 min to remove the precipitation. The absorbance of the supernatant was measured at 510 nm. The hydroxyl radical clearance rate was calculated as follows:

(4) $$\text{Hydroxyl radical clearance rate}\left(\%\right)=\left[1-\frac{A_{1}-A_{2}}{A_{0}}\right]\times 100$$

where *A*_1_ is the absorbance of the sample supernatant, *A*_2_ is the absorbance of the supernatant in which the salicylic acid–ethanol is replaced by the same amount of ethanol, and *A*_0_ is the absorbance of the supernatant in which the sample is replaced by distilled water. The ascorbic acid was used as a positive control.

#### 2.7.2 Ferric reducing antioxidant power (FRAP) activity

The FRAP assay was used to determine the antioxidative of the samples using the method described by Tsai et al with some modifications (Tsai, Huang, & Mau, 2006) and to configure the sample solution with the concentration of 1, 2, 5, 10, and 20 mg/ml. Using 1 ml of sample mixed with 2.5 ml of 0.2 mol/L sodium phosphate buffer (pH 6.6) and 2.5 ml of 1% potassium ferricyanide solution incubated at 50℃ for 30 min, add 2.5 ml of 10% trichloroacetic acid solution, centrifuged at 1,042 *g* for 10 min, and then collected 2.5 ml of supernatant mixed with 2.5 ml of distilled water and 0.5 ml of 0.1% ferric chloride solution. The absorbance at 700 nm was measured after 10 min by a 754 PC spectrophotometer (Shanghai, China). FeSO_4_ aqueous solution was used as positive control.

### 2.8 In vitro digestion simulation

According to the in vitro harmonized protocol described by Minekus (Minekus et al. (2014), the soy peptides were diluted to 10 mg/ml with deionized water and adjusted to pH 2.0 with 1 M HCL. Then, hydrolysis was started by adding the pepsin (2.5 U/mg substrate) from porcine gastric mucosa, the mixture was incubated for 2 hr at 37°C in a water bath with stirring, and pH value was adjusted to 7.2 with 1 M NaOH solution. To simulate the digestion of small intestine, pancreatin (E: S = 4:100) from porcine pancreas was added in the mixture to start the hydrolysis sequentially. The mixture was incubated at 37°C for 2 hr, and the pH value was kept to 7.2 with 1 M NaOH. After the incubation, the digestion process was terminated after enzyme denaturation through increasing the temperature to 95°C for 20 min. The soy peptides were separated from the reaction mixtures after digestion by centrifugation at 8,000 rpm for 10 min. The inhibitory activities of ACE, α‐glucosidase, and DPP‐IV of the supernatants were determined by the methods mentioned above.

### 2.9 Isolation of α‐glucosidase inhibitory peptides from soy protein hydrolysate

#### 2.9.1 Ion‐exchange resin (DEAE‐52)

The soy protein hydrolysate showing highest α‐glucosidase inhibition activity was first fractionated by using anion‐exchange chromatography DEAE‐52. The freeze‐dried hydrolysate was dissolved in distilled water to the concentration of 20 mg/ml. Then, 4 ml of hydrolysate was loaded into the DEAE‐52 anion‐exchange column (6 mm × 30 cm, Qingdao Bishui Hantian Biological Co., Ltd.) pre‐equilibrated with the starting buffer (Tris‐HCl, pH = 8). The adsorbed peptides were eluted as the concentrations of NaCl increased (0, 0.05, 0.1, 0.2, 0.3 M) in the starting buffer with a flow rate of 1.5 ml/min for 6 hr. The elution peaks were monitored at 220 nm, and the fractions were collected every 5 min. Factions eluted within each peak were pooled together, desalted with dialysis bag (200 Da cutoff), and assessed for their α‐glucosidase inhibitory activities. The separation process was repeated for gathering enough to further purification. Then, the sample was concentrated by rotary evaporation and purified by the size‐exclusion chromatography for further analysis.

#### 2.9.2 Gel filtration chromatography (G‐15)

Three microliters of fraction from ion‐exchange chromatography (10 mg/ml) was loaded into a chromatography column (6 mm × 60 cm) packed with the pretreated gel Sephadex G‐15 (Beijing Solarbio Science & Technology Co., Ltd.). Then, the sample eluted using distilled water with the flow rate of 0.5 ml/min. The peptide elution was monitored by measurement of the absorbance at 220 nm, and fractions were collected. Separation process was repeated multiple times, and the α‐glucosidase inhibitory activities of collected fractions were analyzed. The fraction with the α‐glucosidase inhibitory activity marked by H_1‐1_ was concentrated by rotary evaporation and subjected to the subsequent analysis and identification procedure.

### 2.10 Identification of peptide sequence (LC‐MS/MS)

The amino acid sequence and the molecular mass of H_1‐2_were analyzed by (Qingdao Sci‐tech innovation Co., Ltd.) with online nanospray LC‐MS/MS on a Q Exactive Plus coupled to an EASY‐nano‐LC 1200 system (Thermo Fisher Scientific). Five microliters of the sample was loaded into the trap column (Thermo Fisher Scientific Acclaim PepMap C18, 100 μm × 2 cm), with a flow rate of 10 μL/min, and subsequently separated on the analytical column (Acclaim PepMap C18, 75 μm × 15 cm), with a linear gradient, from 3% to 38% B (A: distilled water, B: 0.1% formic acid in ACN) in 60 min. The column flow rate was maintained at 0.5 ml/min with the column temperature of 40°C. The peptide sequences of the major peaks were performed by processing the ion series in MS/MS spectra using Peaks Studio 8.5 and manual interpretation.

A spray voltage of 2 kV was applied, and the instrument, Q Exactive Plus, was operated under switching automatically between MS full scan and data‐dependent fragmentation modes with 27 of collision energy. Ions were scanned at high resolution (70,000 in MS1, 17,500 in MS2), and the MS scan range was 100–1500 m/z at both MS1 and MS2 levels.

### 2.11 Peptide synthesis

Synthetic peptides were provided by Qingdao China Peptides Co., Ltd. using the conventional Fmoc solid‐phase synthesis method. Synthetic peptides were purified and determined by HPLC‐MS analysis with the purification rate over than 98%.

### 2.12 Statistical analysis

The data were expressed as the mean of three replicates and standard deviation (*SD*). Statistical calculation was performed by ANOVA with IBM SPSS statistics, version 25 (IBM Inc.). Differences were considered to be significant at *p* < 0.05.

## 3 RESULTS AND DISCUSSION

### 3.1 Preparation of peptides with α‐glucosidase inhibitory activity

To prepare the antidiabetic bioactive peptides, soy protein isolate (protein content 86.62%) was hydrolyzed with AP, papain, and trypsin, respectively, and α‐glucosidase inhibitory activity of hydrolysates was analyzed.

**Figure 1:**
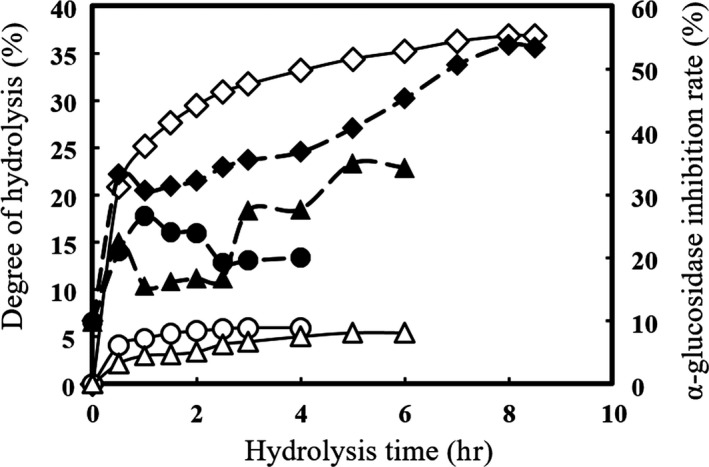
α‐glucosidase inhibition rates (solid symbols) and DH values (open symbols) of hydrolysates prepared with AP ( ♢，♦), papain ( △, ▲), and trypsin (○,●)

As shown in Figure 1, the peptides released by three proteases displayed different hydrolytic processes and different α‐glucosidase inhibitory activities. During all three enzymolysis processes, the DH of hydrolysates increased with hydrolysis time, and the DH of hydrolysates released by papain and trypsin reached 5.92 ± 1.31% and 8.10 ± 0.82%, respectively. The highest DH value of hydrolysates was 36.84 ± 1.20%, which was obtained by AP after 8‐hr hydrolysis time. This result was similar to the study on soy protein hydrolysates by Hrckova et al., and they obtained 35.1% of DH value after 8‐hr hydrolysis time with AP (Hrckova, Rusnakova, & Zemanovic, 2002). It was supposed that different cleavage sites of the three proteases result in the difference of DH value during the hydrolytic processes. AP is an endoenzyme with a broad specificity, which preferentially cleaves peptide bonds on the C‐terminal of hydrophobic amino acid residues, such as Typ, Phe, Leu, Ile, Val, and Met, and higher DH value can be achieved with longer enzymolysis time (Nourmohammadi, SadeghiMahoonak, Alami, & Ghorbani, 2017). While trypsin cleaves solely C‐terminal to Arg and Lys, papain prefers to cleave the peptides bonds between the carboxylic acid group of Lys or Arg and adjacent amino acid residue. The highest α‐glucosidase inhibition rate (53.79 ± 3.24%) of the hydrolysate released by AP was derived at the DH value of 36.84 ± 1.20%. Figure 1 also shows that the DH value positively affected the inhibition rates of the hydrolysates.

Table 1 shows the results of the molecular weight distribution. There were 95.39% of the hydrolysates with molecular weight lower than 2,000 Da and 69.76% with molecular weight lower than 500 Da.

**Table 1:** Molecular weight distribution of soy protein peptides generated by AP enzymolysis

Molecular weight range (Da)	Peak molecular weight	Retention time (min)	Distribution of Mw classes (%)
>5,000	6,764	12.512	1.16
5,000–3,000	3,393	13.851	1.68
3,000–2,000	2019	14.859	2.10
2,000–1,000	1,000	16.223	6.60
1,000–500	500	17.568	18.70
500–180	233	19.049	53.53
<180	179	19.563	16.23

### 3.2 Inhibitory activities of soy protein peptides against α‐glucosidase and DPP‐IV

The soy protein peptides obtained by AP hydrolysis were freeze‐dried after the removal of insoluble precipitation. Inhibitory activities against α‐glucosidase and DPP‐IV of soy protein peptides were measured. α‐Glucosidase is a membrane‐bound enzyme located in the epithelium of the small intestine that catalyses the cleavage of glucose from disaccharides (Matsui et al., 1996). Inhibition of this enzyme has been recognized as an effective approach to reduce the serum glucose level (Johnson et al., 2011). Since hydrolysates are crude mixtures of proteins, the α‐glucosidase inhibitory activity of acarbose (IC_50_ = 0.52 ± 0.05 mg/ml) was higher than the hydrolysates that the IC_50_ of α‐glucosidase inhibitory activity was 4.94 ± 0.07 mg/ml.

DPP‐IV is known for its inactivation of incretin hormones GLP‐1 and GIP (Drucker, 2007). By inhibiting DPP‐IV, it is possible to increase the half‐life of GLP‐1 and GIP resulting in enhanced insulin secretion (Nongonierma & FitzGerald, 2013). The same preparation had high DPP‐IV inhibition activity with the IC_50_ value of 2.73 ± 0.08 mg/ml.

The soy peptides we screened contained small peptides with a molecular weight between 180 and 500 Da, that is, about 2–5 amino acids.

### 3.3 Antioxidation and ACE inhibition activities of soy protein hydrolysates

**Figure 2:**
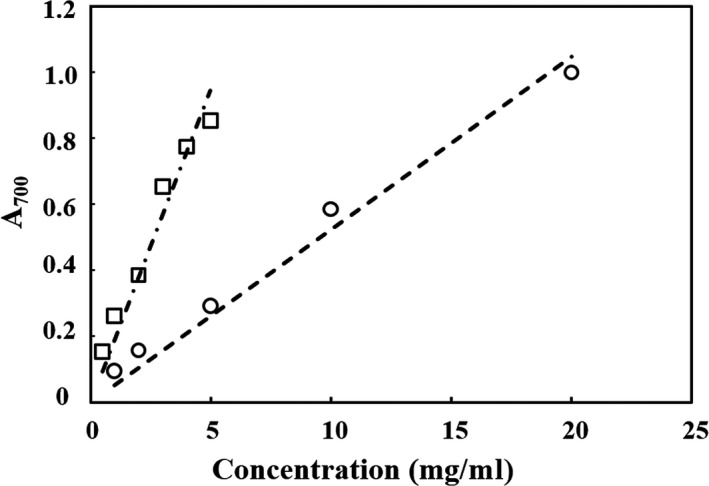
Ferric reducing antioxidant power with different concentrations of peptides solution (○) and FeSO_4_ solution (□)

Since oxidative stress also affects the development of diabetes, the antioxidative activities of hydrolysate were evaluated. The soy hydrolysates obtained by AP exhibited hydroxyl radical scavenging ability (IC_50_ = 5.30 ± 0.15 mg/ml), while it was weaker than that of vitamin C (IC_50_ = 0.54 ± 0.04 mg/ml). As shown in Figure 2, iron ion reduction ability of both hydrolysates and FeSO_4_ solution was linearly related to concentration, and at the same concentration, ferrous sulfate possessed stronger reduction ability.

Renin–angiotensin system is an important regulator of blood pressure and vascular distention, and the ACE plays a key role by hydrolyzing angiotensin I to angiotensin II, which possesses strong vasoconstrictor effect. The soy protein hydrolysate produced by AP inhibited 20.31 ± 2.37% of ACE that was in accordance with the study performed by Wu and Ding (2002). Therefore, soy protein hydrolysates showed the potential to be a multifunction nutraceutical agent. The structure of the peptides may be closely related to ACE inhibitory effects. It is reported that the position of a hydrophobic amino acid at the C‐terminal end is associated with ACE inhibitory activity (Escudero, Toldra, Sentandreu, Nishimura, & Arihara, 2012). Soy protein belongs to plant protein and contains more hydrophobic amino acids (Zhang et al., 2017).

### 3.4 Simulated gastrointestinal digestion

**Figure 3:**
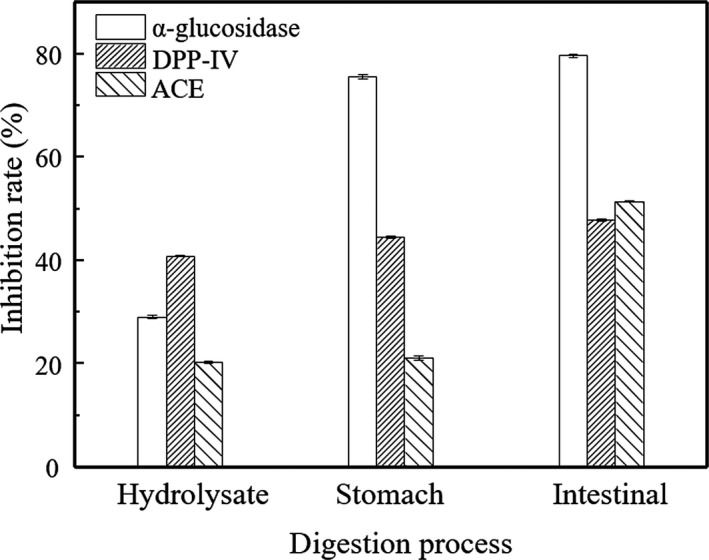
Inhibitory activities of peptides against α‐glucosidase, DPP‐IV, and ACE during the digestion process

Before and after in vitro simulated digestion, the inhibitory activities against α‐glucosidase, DPP‐IV, and ACE of soy protein peptides were measured, and the results are shown in Figure 3. Before digestion, soy peptides with a concentration of 10.0 mg/ml indicated that the inhibition rate was 29.04 ± 1.49%, 40.85 ± 0.82%, and 20.31 ± 2.37% against α‐glucosidase, DPP‐IV, and ACE, respectively. After gastric phase digestion, α‐glucosidase inhibitory activity of the peptides significantly increased up to 75.53 ± 1.44% (*p* < 0.05), and after intestinal digestion, it reached to 77.64 ± 1.07%. For inhibitory activities against DPP‐IV, compared with undigested soy peptides, we also observed significant change (*p* < 0.05) after gastric and intestinal digestion, and it was 44.51 ± 0.95% and 47.94 ± 1.10%, respectively. For ACE inhibition activity, we found that after gastric digestion, it increased slightly to 21.71 ± 1.52% (*p* > 0.05), and after intestinal digestion, the inhibition rate enhanced significantly to 51.43 ± 1.74% (*p* < 0.05). Akillioglu and Karakaya reported an increase of ACE inhibitory activity in legume hydrolysates after the gastrointestinal digestion (Akıllıoğlu & Karakaya, 2009). Conversely, Tavares et al found that a slight decrease in ACE inhibitory activity of peptides from whey protein after the digestion (Tavares et al., 2011). Although we do not know the reasons leading to different results, the amino acid sequence of peptides and inhibitory mechanism of peptides against ACE should be considered.

In general, this result demonstrated that the peptides could endure the whole digestive environment and still maintain or enhance their bioactivity.

### 3.5 Separation and purification

**Figure 4:**
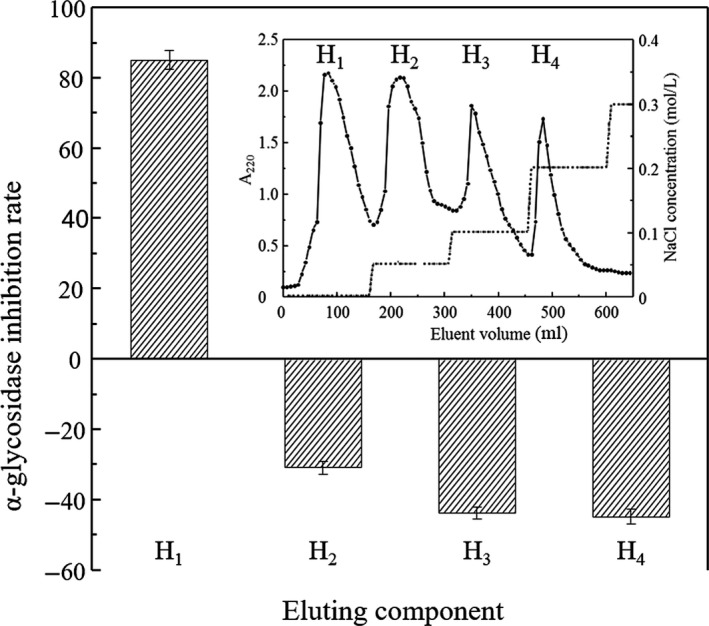
α‐glucosidase inhibitory activities of fractions H_1_, H_2_, H_3_, and H_4_. Inset: Elution profile of hydrolysates with DEAE‐52

In order to purify peptides with α‐glucosidase inhibitory activity, successive chromatographic separations were performed with anion‐exchange resin DEAE‐52 and Sephadex‐15 gel. As shown in Figure 4 (inset), four fractions (H_1_, H_2_, H_3_, and H_4_) were obtained after gradient elution through DEAE‐52. α‐glucosidase inhibition rates of each fraction were determined, and the results were shown in Figure 4. After the H_1_ was washed off by distilled water, it was found that H_1_ was only the effective inhibitor on the α‐glucosidase with inhibition rate 87.10 ± 2.70%, and the inhibitory activity was higher than the original hydrolysates. All other fractions were not able to inhibit α‐glucosidase.

**Figure 5:**
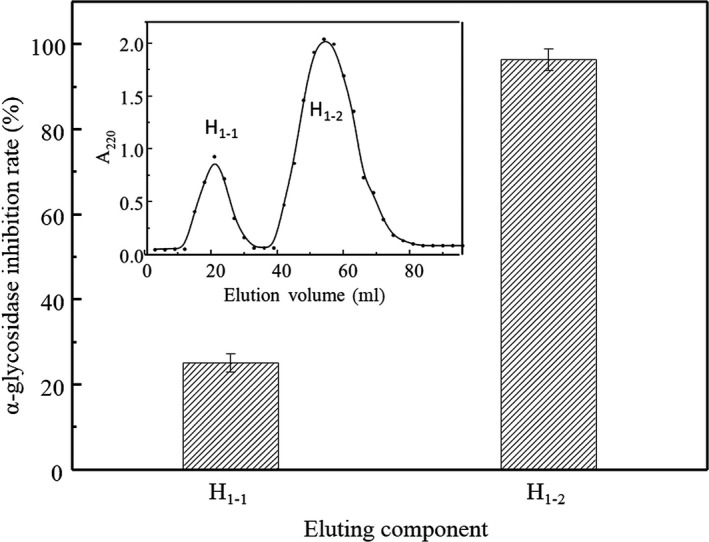
α‐glucosidase inhibitory activities of fractions H_1‐1_, H_1‐2_. Inset: Elution profile of H_1_ with Sephadex G15

We collected and concentrated H_1_ fraction by rotary evaporation, then purified further by size‐exclusion chromatography. As shown in Figure 5 inset, fractionation by size of the H_1_fraction revealed a relatively clear molecular weight distribution profiles with only two main peaks (H_1‐1_, H_1‐2_). Figure 5 illustrates these two fractions both had α‐glucosidase inhibition activity, and H_1‐2_ showed high α‐glucosidase inhibition activity up to 95.35 ± 2.70% inhibitory rate, which was much higher than H_1‐1_ with 26.08 ± 2.12% inhibitory rate.

### 3.6 Identification of purified peptides

**Figure 6:**
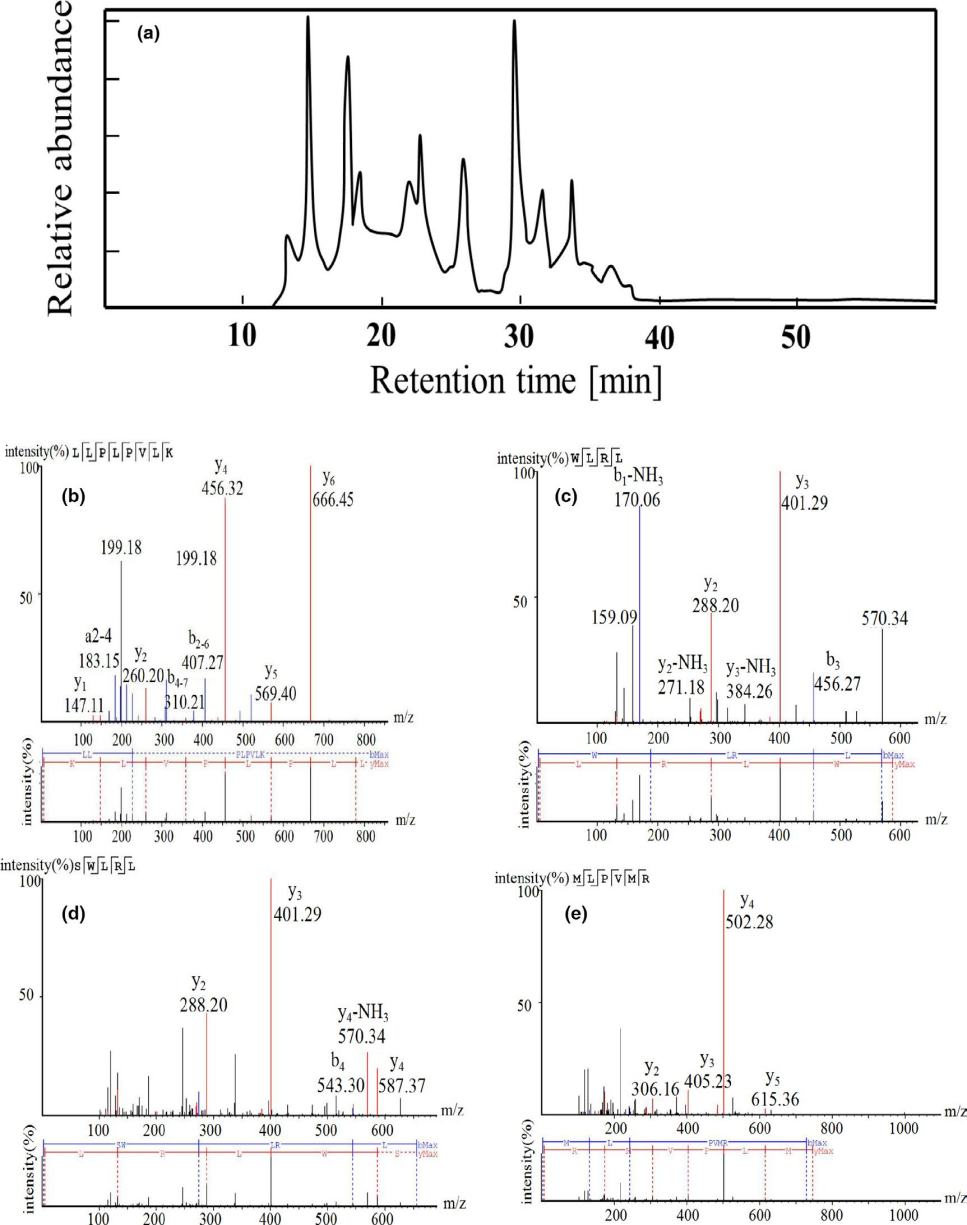
Identification of the peptides by EASY‐MS/MS. (a) HPLC chromatography of peptides; (b) LLPLPVLK; (c) WLRL; (d) SWLRL; (e) MLPVMR

We determined molecular weight of the purified fraction H_1‐2_with EASY‐MS. The results showed four abundant ions with m/z of 893.6, 588.4, 675.4, and 745.4, which revealed four oligopeptides that would be further sequenced using LC‐MS/MS. We obtained the complete amino acid sequences by amino acid composition analysis and manual calculation of the MS/MS spectrum. As shown in Figure 6, the first octapeptide was identified as LLPLPVLK, while the second peptide consisted of four amino acids with the sequence of WLRL. Besides, peptides with five amino acid SWLRL and hexapeptide MLPVMR were also identified. Compared with the previously reported hypoglycemic peptides, such as N159‐1 (PFP separated from *Aspergillus oryzae*) (Kang, Yi, & Lee, 2013), albumin (KLPGF) (Yu, Yin, Zhao, Liu, & Chen, 2012), and egg white protein (RVPSLM) (Yu et al., 2011), there appear to be no primary structural homology to the four oligopeptides we found. It is not difficult to point out that hydrophobic amino acids were common in these hypoglycemic peptides. In particular, Leu and Pro showed outstanding contributions to these oligopeptides separated from soy protein (Sadri, Larki, & Kolahian, 2017). It was reported that Pro and Leu in peptides were considered as vital amino acids playing inhibition effect against α‐glucosidase separately or synergistically, which should be taken into account in the synthesis of novel α‐glucosidase inhibitory peptides for antidiabetic treatment.

### 3.7 Antidiabetic properties of synthetic peptides

The synthesized peptides have been confirmed the great inhibitory effect on α‐glucosidase activity. The hexapeptides, MLPVMR, did not show inhibitory activity. However, other three peptides, LLPLPVLK, SWLRL, and WLRL, had great inhibitory influence on α‐glucosidase with the IC_50_ value of 237.43 ± 0.52, 182.05 ± 0.74, and 165.29 ± 0.74 μmol/L. It has been suggested that the inhibitory mechanism could be through the hydrophobic interactions of the polypeptide with the active site of the enzyme, as it has been reported for other inhibitors (Bharatham, Bharatham, Park, & Lee, 2008). The peptides we found are rich in hydrophobic amino acids, especially the Leu, which might play an important role to inhibit α‐glucosidase.

## 4 CONCLUSIONS

In summary, the peptides possessing high α‐glucosidase inhibitory effect were successfully prepared from soy protein treated via an AP process at the DH value of 36.84 ± 1.20% in the present study. The soy protein peptides also exhibited antioxidative activity and inhibition activities against DPP‐IV and ACE. The peptides with α‐glucosidase inhibition activity were separated with anion‐exchange resin, Sephadex G‐15 gel, which was determined using LC‐MS/MS sequentially. The IC_50_ α‐glucosidase inhibitory activity values of LLPLPVLK， SWLRL, and WLRL were 237.43 ± 0.52, 182.05 ± 0.74, and 165.29 ± 0.74 μmol/L, respectively. Compared with other α‐glucosidase inhibitory peptides, these three peptides we found showed different structural homology from them, as well as lower IC_50_ value than acarbose. Therefore, this research can be referenced for the development of novel antidiabetic peptide nutraceuticals. Also, in order to validate the findings of the present study, further research in cell culture and in vivo should be needed.

## CONFLICT OF INTEREST

The authors declare that they do not have any conflict of interests.

## ETHICAL STATEMENT

Human testing and animal testing were unnecessary in this study.
